# Human Lung Cancer Cell Line A-549 ATCC Is Differentially Affected by Supranutritional Organic and Inorganic Selenium

**DOI:** 10.1155/2014/923834

**Published:** 2014-11-12

**Authors:** Lérida Liss Flores Villavicencio, Gustavo Cruz-Jiménez, Gloria Barbosa-Sabanero, Carlos Kornhauser-Araujo, M. Eugenia Mendoza-Garrido, Guadalupe de la Rosa, Myrna Sabanero-López

**Affiliations:** ^1^Universidad de Guanajuato, Lascurain de Retana 5, 36000 Guanajuato, GTO, Mexico; ^2^Departamento de Fisiología, Biofísica y Neurociencias, CINVESTAV, Avenida Instituto Politécnico Nacional 2508, San Pedro Zacatenco, Gustavo A. Madero, 07360 Ciudad de México, DF, Mexico

## Abstract

The effects of organic and inorganic forms of selenium (Se) on human cells have been extensively studied for nutritional concentrations; however, to date, little is known about the potential toxicity at supranutritional levels. In the present study we determined the effects of sodium selenite (SSe) and selenomethionine (SeMet) on cell growth and intracellular structures in lung cancer cells exposed at Se concentrations between 0 and 3 mM. Our results showed that SSe affected cell growth more rapidly than SeMet (24 h and 48 h, resp.). After 24 h of cells exposure to 0.5, 1.5, and 3 mM SSe, cell growth was reduced by 10, 50, and 60%, as compared to controls. After 48 h, nuclear fragmentation was evident in cells exposed to SSe, suggesting an induction to cell death. In contrast, SeMet did not affect cell proliferation, and the cells were phenotypically similar to controls. Microtubules and microfilaments structures were also affected by both Se compounds, again SSe being more toxic than SeMet. To our knowledge, this is the first report on the differential effects of organic and inorganic Se in supranutritional levels in lung cancer cells.

## 1. Introduction

Selenium (Se), as part of selenoproteins, plays a major role in the metabolism of thyroid hormones and cellular protection against oxidative stress [1, 2]. Selenoenzymes are needed for fetal cell differentiation, growth, and development [3] and Se deficiency results in decreased immunoglobulin production and activity of glutathione peroxidase, which reduces cell hydroperoxides [4]. Debate remains on the minimum required Se concentration in plasma for maximal expression of a variety of enzymes. For glutathione peroxidase 75 ng/mL is required, which can be achieved with an intake of 40 *μ*g/d Se [5–7]. Selenoprotein activity maximizes with plasma Se levels between 1.2 and 1.7 *μ*mol/L [8]. According to a study performed by Thomson [9], selenium concentration that can be achieved in blood to an optimum metabolism might depend on population type. The main sources for Se are cereals, meat, and fish, with milk and eggs providing smaller amounts [10, 11].

The effects of Se in living systems depend on the chemical structure of its compounds, which are in turn related to their concentration and bioavailability. For example, the bioavailability of selenomethionine (SeMet) is greater than that of sodium selenite (SSe) [12].

Even when high Se doses may be toxic [13, 14], supranutritional levels of this element exert protective effects against some diseases including hypertension and cancer [15–19]. Although the anticarcinogenic effects of Se have been demonstrated [20] to our knowledge, few studies compare the effects when different chemical forms of Se are supplied at supranutritional levels.

The present study was conducted by exposing human cancer cell line A-549ATCC to supranutritional organic and inorganic Se (up to 3 mM) in order to determine the effect on cell growth, DNA, and cytoskeletal structures. Our results suggest that Se, administered at supranutritional concentrations to human lung cancer cells, can be further explored as an option for cancer treatment. The delivery might be performed via functionalized nanoparticles.

## 2. Materials and Methods

### 2.1. Chemicals

Seleno-L-methionine (SeMet) and sodium selenite (SSe) were purchased from Sigma (St. Louis, MO, USA). All other chemicals used in this study are commercially available.

### 2.2. Cell Culture

The human lung cancer cell line A-549 ATCC was selected for this study. The cells were cultivated in D-MEM (Dulbecco's Modified Eagle's Medium, GIBCO, USA) supplemented with fetal bovine serum 10% (GIBCO, USA) and incubated at 37°C, 5% CO_2_.

### 2.3. Exposure of A549 Cells to Selenium Compounds

Subconfluent cultures were prepared in 12-well plates (Costar, Corning, USA), which were exposed to either SSe or SeMet at Se concentrations of 0.5, 1.5, and 3 mM for 24 h. After exposure, the cell viability was determined by trypan blue. The experiments were performed in triplicate and the results reported as means ± standard deviation (SD). Statistical analysis was performed by one-way analysis of variance. Tukey HSD (Honestly Significant Difference) was used as a* post hoc* test to determine significant differences between treatments (*P* < 0.05) and Minitab 17.0.1 software was used to perform data analysis.

### 2.4. Protein Profile Analysis

A549 cells were exposed to selenium compounds at a concentration of 0.5 mM for 24 h. Later, the cells were washed three times with PBS, solubilized with 2% SDS plus inhibitor proteases cocktail (Roche, Germany). The samples (40 *μ*g/lane) were fractionated in SDS-PAGE 10% gel under reducing conditions [21] and stained with Coomassie Blue 0.25% (Sigma, USA). Protein standards were used for reference (Sigma, USA).

### 2.5. Immunocytochemistry Analysis

Control cells and those exposed to selenium compounds were fixed with 4% p-formaldehyde for 20 min, permeabilized with 0.05% Triton buffer containing 10 mM Tris, 5 mM KCl, and 1 mM MgCl_2_, and later exposed to anti-*β*-Tubulin/anti-mouse IgG-FITC antibodies or FITC-phalloidin (Sigma Aldrich). The preparations were mounted using Vecta Shield-DAPI (Vector Laboratories, USA) and observed under a fluorescence microscope (Leica, DMLS) using a 450–490 nm B filter.

### 2.6. DNA Preparation

The cells were exposed to selenium compounds as previously described. To perform the extraction of the cell culture, the medium was removed and 300 *μ*L of lysis buffer was added. Genomic DNA extraction was performed according to Sambrook et al. [22]. DNA samples were analyzed on 1.5% agarose gels and stained with ethidium bromide. The bands were visualized with UV light at 260–280 nm (Gene Genius, Syngene).

## 3. Results

### 3.1. Effect of Organic and Inorganic Selenium on Cell Growth

**Figure 1:**
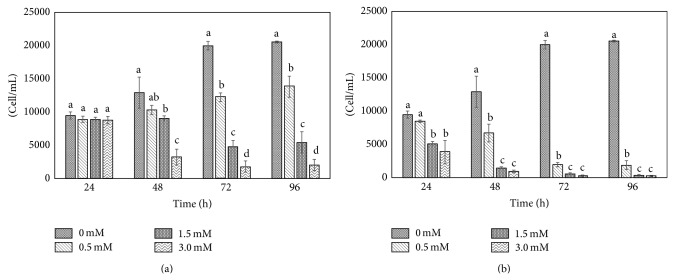
Cell growth of lung cancer cells exposed to 0; 0.5; 1.5; and 3.0 mM Se supplied as (a) SeMet or (b) SSe for 96 h. Values are the arithmetic mean ± standard deviation (*n* = 3). Different letters show significant differences in cell growth between each concentration at the same time (*P* < 0.05).

One-way analysis of variance was used to perform data analysis on cell growth. Tukey HSD (Honestly Significant Difference) was used as a* post hoc* test to determine significant differences between means in the different treatments (*P* < 0.05) and Minitab 17.0.1 software was used to perform data analysis. Cell growth of lung epithelial cells exposed to SeMet and SSe is shown in Figures 1(a) and 1(b), respectively. At time 0 h, every treatment was started with 6,165 cells/mL.  Figure 1(a) shows no difference in cell growth between treatments (SeMet) at 24 h of exposure. However, after this point, significant differences (*P* < 0.05) in cell growth with respect to controls were observed, regardless of the concentration. At 48 h, Se levels of 0.5, 1.5, and 3 mM reduced cell growth reduction were in 10, 30, and 70%, respectively (*P* < 0.05). The growth inhibition of SeMet after 96 h was similar to that at 72 h, with values about 30, 70, and 90%.

Differential results were obtained in lung epithelial cells treated with SSe (Figure 1(b)). Growth inhibition was observed at 24 h where 1.5 and 3.0 mM Se significantly (*P* < 0.05) decreased cell growth with respect to control by 45 and 60%, respectively. After 24 h, a trend in cell growth decrease was observed in every Se concentration supplied as SSe. Moreover, at 72 and 98 h, the presence of SSe provoked a decrease of cell growth of more than 90%. Thus, SSe exerted more toxic effects to cells than SeMet.

### 3.2. Protein Profile Analysis

**Figure 2:**
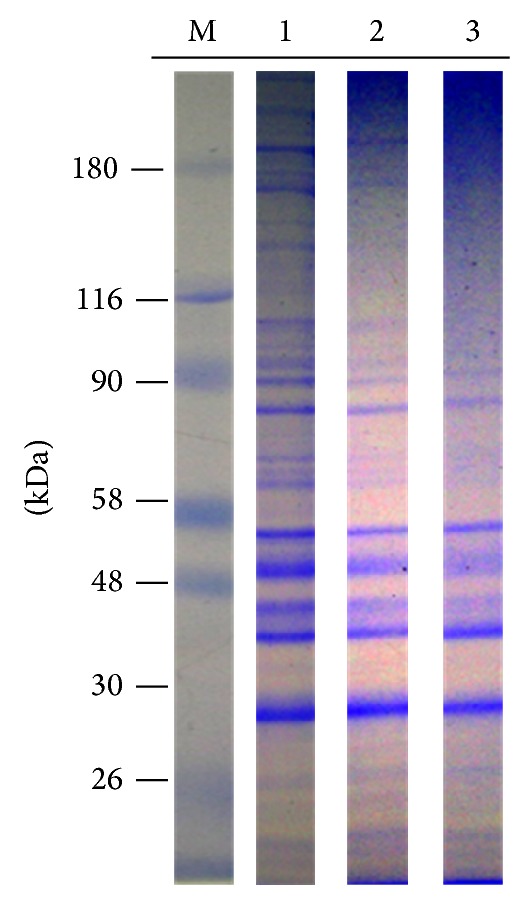
Protein profile of cells exposed to SeMet and SSe: controls (Lane 1) cells treated with 0.5 mM SeMet (Lane 2) and SSe (Lane 3) for 48 h; standard molecular mass markers (M) are shown in the first lane.

Figure 2 shows the peptide profiles of control cells (Lane 1) and those treated with 0.5 mM Se supplied as either SeMet (Lane 2) or SSe (Lane 3). As observed in this figure, in controls (Lane 1), the major peptides with MW ≥ 190, 170, 150, 110, 90, 80, 65, 60, 55, 48, 40, 35, and 28 kDa are present. In contrast, the profiles of cells treated with SeMet and SSe show that peptides with MW ≥ 60, 65, 150, 170, and 190 kDa decrease under SeMet and SSe treatment.

### 3.3. Nuclei Analysis

**Figure 3:**
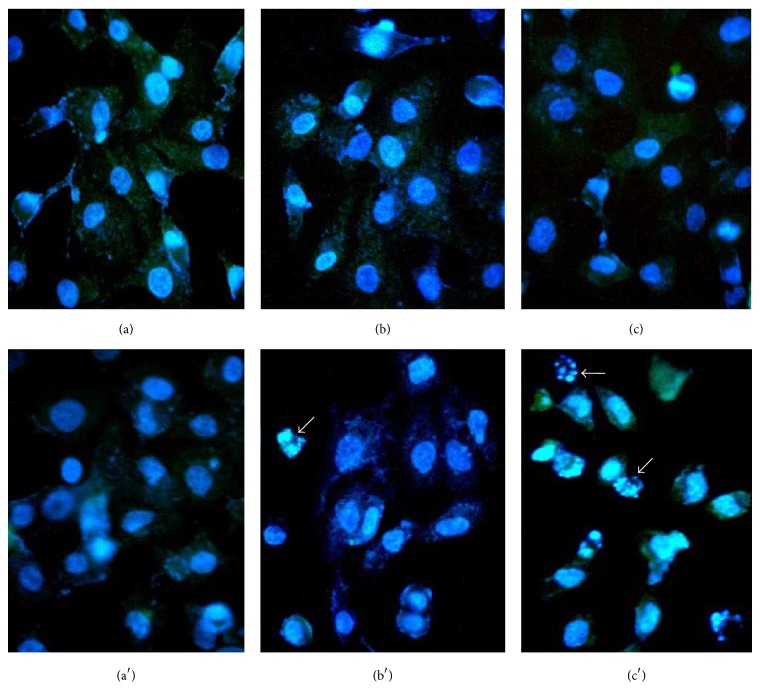
Nuclei of cells exposed to SeMet and SSe: controls (a and a′); cells exposed to 0.5 and 1.5 mM Se as SeMet (b, c); cells exposed to 0.5 and 1.5 mM Se as SSe (b′, c′) for 48 h. Arrows indicate nuclear fragmentation in SSe-treated cells.

**Figure 4:**
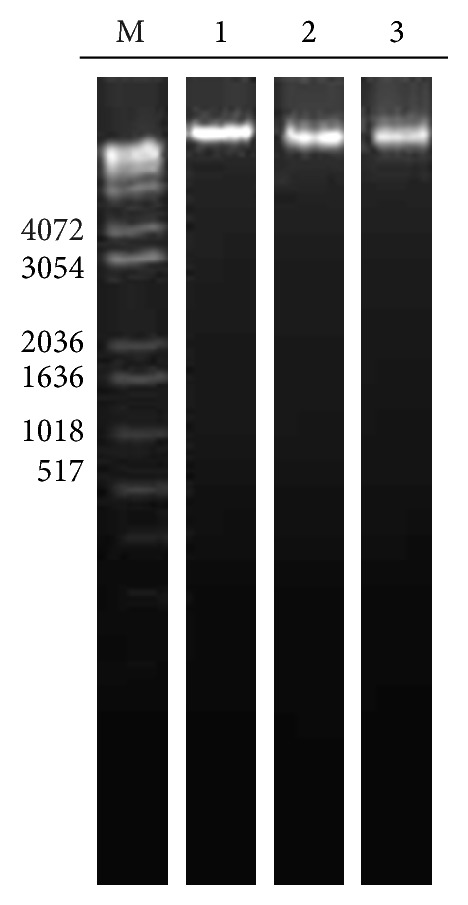
Genomic DNA of cells exposed to SeMet and SSe. M corresponds to the molecular markers (bp); controls (Lane 1); cells exposed to 0.5 mM Se as SeMet (Lane 2); cells exposed to 0.5 mM Se as SSe (Lane 3).

The nuclei of cells were stained with 4′,6-diamidino-2-phenylindole (DAPI). In control nucleus (Figures 3(a) and 3(a′)) was observed at fluorescence pattern homogeneous. Similar results were observed in the nuclei of cells treated with 0.5 and 1.5 mM Se given as SeMet (Figures 3(b) and 3(c)). In contrast, some the nuclei of cells treated with 0.5 and 1.5 mM Se in the form of SSe showed nuclear fragmentation (Figures 3(b′) and 3(c′)). Results indicate that damage at the nuclear level by the SSe is dependent on concentration and suggests cell death induction. However, the genomic analysis of DNA (Figure 4) showed no alterations of this structure after treatment with either organic or inorganic selenium.

### 3.4. Microtubule Distribution

**Figure 5:**
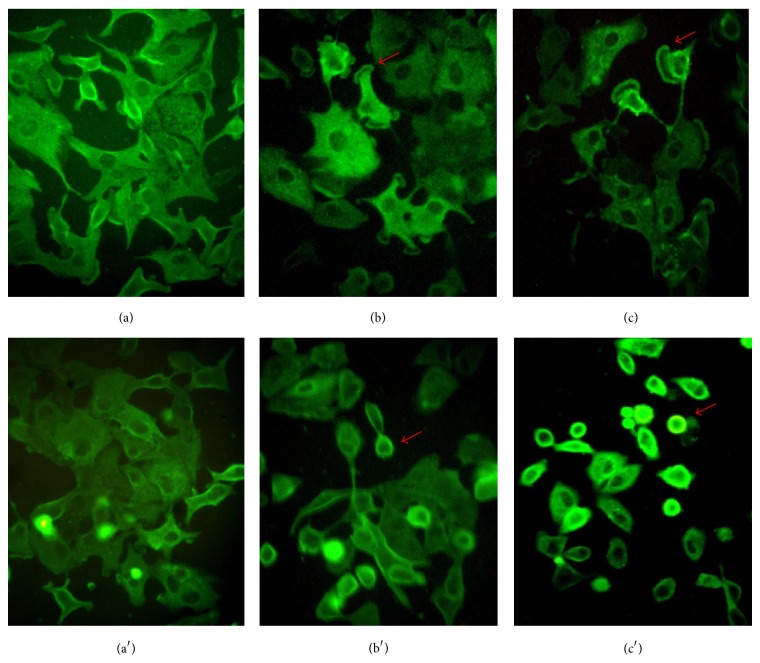
Microtubules of cells exposed to SeMet and SSe. Controls (a and a′); cells treated with 0.5 and 1.5 mM Se as SeMet (b and c) and SSe (b′, c′) for 48 h. Arrows show changes in microtubule distribution and loss of morphology.

Microtubule distribution in cells treated with both selenium compounds was analyzed by immunocytochemistry after 48 h of exposure. Tubulin staining showed in control cells a microtubule pattern, where the microtubule network in the cytoplasm outlines the cell morphology (Figures 5(a) and 5(a′)).

The cells treated with 0.5 and 1.5 mM Se as SeMet (Figures 5(b) and 5(c)) showed that the fluorescence distribution is altered on the edges of the cell membrane. This alteration is more evident in cells exposed to the 1.5 mM level (Figure 5(c)). A loss of fluorescence is also observed, in addition to cell morphology alterations.

The distribution of the microtubule network in cells treated with 0.5 and 1.5 mM Se as SSe (Figures 5(b′) and 5(c′)) showed a severe alteration of the fluorescence patterns compared to control cells (Figures 5(a) and 5(a′)). The concentration-dependent loss of morphology is evident (Figure 5(c′)) and there are a large number of rounded cells that retain fluorescence around the perinuclear region. The results indicate that the microtubular network was affected by both SeMet and SSe; however, this network is even more affected in cells exposed to SSe.

### 3.5. Microfilament Distribution

**Figure 6:**
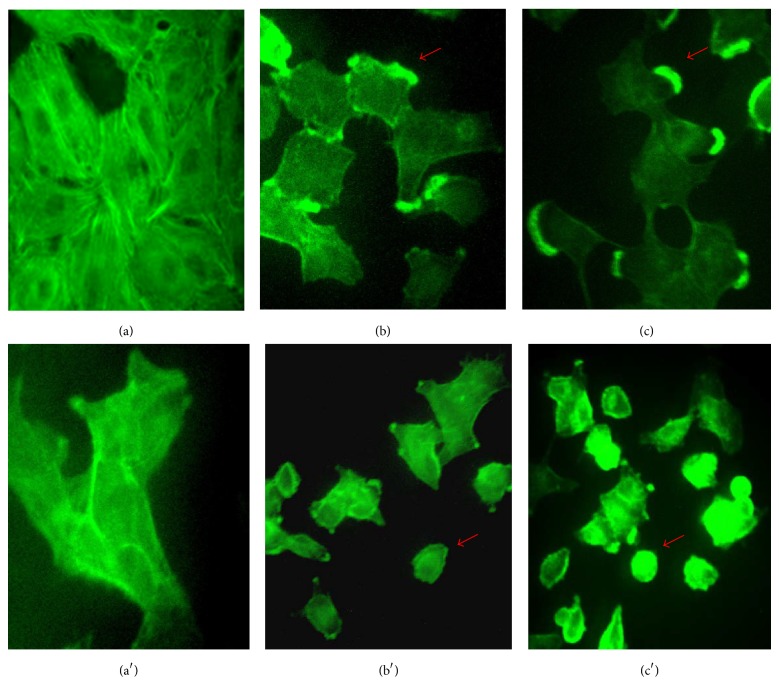
Microfilaments of cells exposed to SeMet and SSe. Controls (a and a′); cells treated with 0.5 and 1.5 mM Se as SeMet (b and c) and SSe (b′, c′), respectively, for 48 h. Arrows point to the fluorescent plates at the edges of the membrane and loss of spreading morphology.

The actin microfilaments distribution was examined using phalloidin, which specifically binds to polymerized actin or F-actin. Control cells (Figure 6(a)) show typical microfilament bundles in the cytoplasm towards the plasma membrane. Fluorescence is also observed at the edges of intercellular junctions (Figure 6(a′)). In cells exposed to SeMet at 0.5 and 1.5 mM Se, fluorescent plaques appeared at the edges of the membrane (Figures 6(b) and 6(c)). In cells exposed to SSe at the same Se concentrations, the fluorescence showed to be distributed in the cytoplasm (Figures 6(b′) and 6(c′)). No filamentous structures were observed and loss of extended cell shape was evident. Our results indicate that SeMet and SSe alter the microfilament arrangement, affecting cell morphology integrity in a concentration-dependent manner.

## 4. Discussion

The effects of inorganic Se, selenite, and selenate on cancer development have been widely analyzed [23, 24]. To a lesser extent, anticarcinogenic Se activity has been explored. Among the organoselenium compounds, SeMet has been extensively used to evaluate the anticarcinogenic activity because it is the main natural form of this element in foods [11]. However, few studies compare the performance of organic and inorganic Se.

Elemental speciation is strongly related to bioavailability and metabolic transformation. In the case of Se, the absorption of SSe (the inorganic form) is lower than that of SeMet (the organic form) [25]. High concentrations of SeMet produce toxic effects similar to those induced by inorganic Se [13].

Our results indicated that the effects of organic and inorganic forms of selenium on lung cancer cells were concentration and speciation dependent. Both compounds inhibited cell growth and induced changes in cytoskeleton organization and protein expression. In this context, the decrease of high molecular weight proteins can be the microtubules binding protein (MBP) and/or the actin binding protein (ABP) [26]. In particular, the results evidence a dramatic change of fluorescent actin fibers consisting of F-actin patches decorating by FITC-phalloidin. In this regard, some ABP may be involved in the nucleation of these patches, as actinogelin [27] which induces gelation of F-actin; the protein consists of subunits of 112,000–115,000 daltons. Alternatively, other ABP as vinculin or *α*-actinin can be involved [28]. This aspect is open to research.

In general, SSe produced more pronounced effects. Even when SSe is expected to be less bioavailable than SeMet, our results indicated that, at a same Se concentration, SSe was more toxic to the cells.

The essential nutritional importance of Se is due to its antioxidant action through enzymes such as glutathione peroxidase and thioredoxin reductase involved in the protection against damage produced by reactive oxygen species [2, 24]. Cells adequately supplied with Se are less susceptible to the damage effects of endogenously or exogenously generated oxygen radicals, which may attack cellular DNA. These situations are valid when dealing with the preventive activity of Se. In our study, we used lung cancer cells. Our results on the DNA integrity showed no difference in DNA structure when cells were exposed to 0.5 mM Se supplied as either SeMet or SSe.

Staurosporine (St) is a competitive inhibitor of protein kinases that binds to kinases with high affinity and little selectivity [29]. St has been considered a valuable tool for the study of apoptosis, in several cell types that implicate changes in cell morphology from a flat to a stellate shape and nuclear fragmentation [30–33]. For all the above, the St was used as control to support our results of nuclear fragmentation (Figure 3).

The results of the cells exposed to 20 uM staurosporine for 20 h show nuclear fragmentation (DAPI) and morphological changes characteristic of apoptosis with apoptotic bodies and altered distribution blebs actin (panel A: Figures 1(b) and 1(b′), see Supplementary Material available online at http://dx.doi.org/10.1155/2014/923834).

Analysis of the genomic DNA shows DNA integrity and ribosomal RNA degradation compared to control cells not exposed to staurosporine (Supplementary Material, Figure 1(b)). However, 90% of the cells showed fragmentation (Supplementary Material, Figure 2(a)), unchanged at electrophoretic pattern of DNA that usually occurs in classical apoptosis. Given this, the results of nuclear fragmentation and DNA integrity with SSe and SeMet are not “artifacts” (Figures 3 and 4).

This reflects that classical apoptosis is not present, despite the fact that the inherent morphological alterations of an apoptotic cell, that is, altered distribution of actin, formation blending, alternatively can be induced by antiapoptotic molecules and activate mechanisms to recover cell homeostasis [34].

Our results differ with those reported by Menter et al. [35] who observed DNA fragmentation of prostate cancer cells treated with SeMet or SSe. This provides additional evidence on the dependence of Se action on cell type.

The high cytotoxicity of inorganic Se reported in the literature [36] is also observed in this study. Our results do not agree in the case of SeMet, the organic compound, which only showed minor cytoskeleton reorganization.

Cellular structures such as microtubules and microfilaments are involved in vital functions such as motility, secretion, and mitosis. Thus, the dynamics of these structures are essential in normal cell physiology [37–39]. For this reason, a variety of antitumor agents with action on microtubules have been developed [20]. The results of our study indicated that both SeMet and SSe affected the microtubular and microfilament network. Damage is more evident in cells exposed to SSe. Shi et al. [20] demonstrated that SSe disrupted microtubule assembly on leukemic HL60 cells but no data was found on the effects of SeMet on this type of cell.

According to our results, SSe may be used for the therapeutic treatment of cancer; however, one challenge would be to determine the proper via for SSe delivery to target cells. Recently, in glioma cell line, the alkylating agent temozolomide (TMZ) conjugated with selenium increased its potential as anticancer agent [40]. Moreover, chitosan stabilized selenium nanoparticles (Ch-Se NPs) were studied for this purpose. Estevez et al. [40] compared the effects of Ch-Se NPs and other Se compounds on hepatocarcinoma cells. According to their results, Ch-Se NPs are a novel compound for future applications as chemotherapeutic agent. Thus, the SSe may be delivered as NPs; however, further investigation should be performed.

## 5. Conclusions

This study showed a comparison among the effects of organic (SeMet) and inorganic Se (SSe) at supranutritional concentrations on lung epithelial cells. Inorganic Se was more effective in reducing cell growth and mechanism of action involving proteins, nuclear, microfilaments, and microtubule damage than those provoked by SeMet, even when SeMet is more bioavailable than SSe. If intended for the therapeutic treatment of cancer, SSe may be delivered through targeted nanoparticles directed to cancer cells.

## Supplementary Material

Staurosporine (St) was used as control to support our results of nuclear fragmentation. The results of the cells exposed to 20 *µ*M staurosporine for 20 h, show nuclear fragmentation (DAPI) and morphological changes characteristic of apoptosis with apoptotic bodies, and altered distribution blebs actin (panel A: Fig 1b and b'. Supplementary material). Analysis of the genomic DNA shown DNA integrity and ribosomal RNA degradation compared to control cells not exposed to staurosporine (Supplementary Material, Fig. 1B). However, there is 90% cells with nuclear fragmentation (Supplementary Material, Fig. 2a), unchanged at electrophoretic pattern of DNA that usually occurs in classical apoptosis. All this supports, the results of nuclear fragmentation and DNA integrity with SSe and SeMet are not “artifacts” (Fig. 3 and 4 manuscript).
