# Supplementary material for: Human Lung Cancer Cell Line A-549 ATCC Is Differentially Affected by Supranutritional Organic and Inorganic Selenium
Source: Bioinorg Chem Appl. 2014 Nov 12;2014:923834. doi: 10.1155/2014/923834 (PMC4244949; doi:10.1155/2014/923834)
Supplement: Supplementary file 1 — Staurosporine (St) was used as control to support our results of nuclear fragmentation. The results of the cells exposed to 20 µM staurosporine for 20 h, show nuclear fragmentation (DAPI) and morphological changes characteristic of apoptosis with apoptotic bodies, and altered distribution blebs actin (panel A: Fig 1b and b'. Supplementary material). Analysis of the genomic DNA shown DNA integrity and ribosomal RNA degradation compared to control cells not exposed to staurosporine (Supplementary Material, Fig. 1B). However, there is 90% cells with nuclear fragmentation (Supplementary Material, Fig. 2a), unchanged at electrophoretic pattern of DNA that usually occurs in classical apoptosis. All this supports, the results of nuclear fragmentation and DNA integrity with SSe and SeMet are not “artifacts” (Fig. 3 and 4 manuscript). [file 923834.f1.pdf]

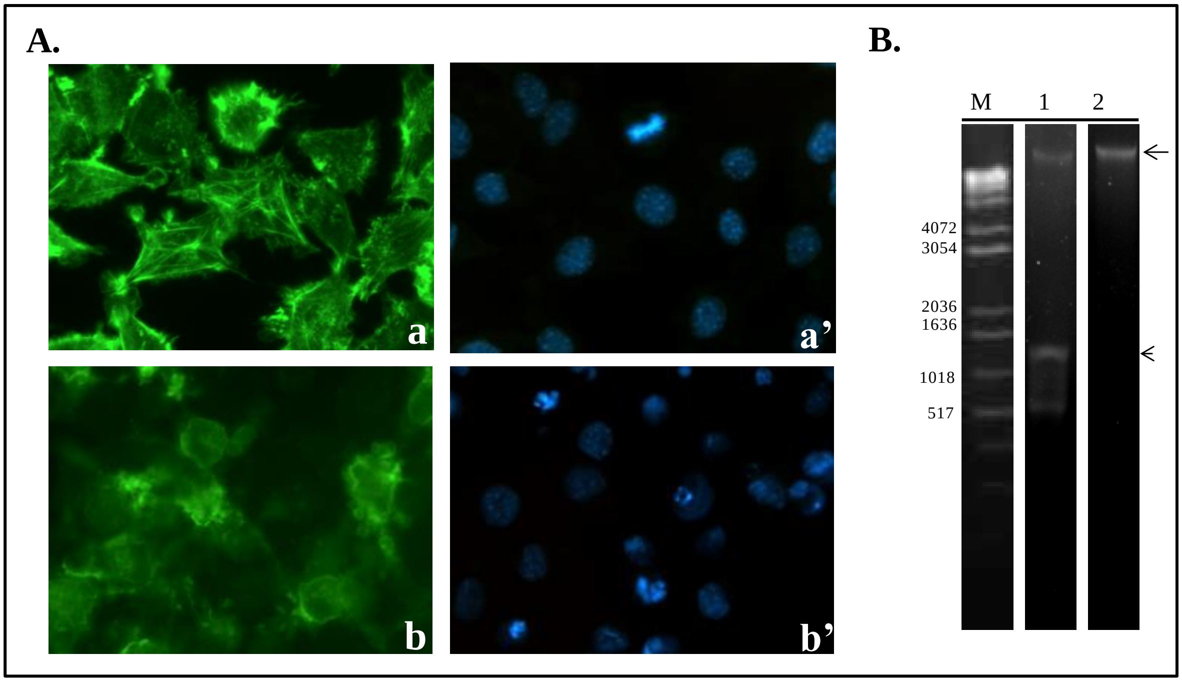

Figure 1. Effect of staurosporine on epithelial cells (panel A). The actin cytoskeleton (a) and the nuclei (a ') in control cells and exposed to staurosporine 20μM at 20h (b and b'). Microfilaments were decorated with FITC-phalloidin, and the DNA was stained with DAPI. Panel B. The genomic DNA of control cells (lane 1) and treated with staurosporine (lane 2) were observed, lane M is size markers (bp). Electrophoresis was performed on a gel of 1.5% agarose.

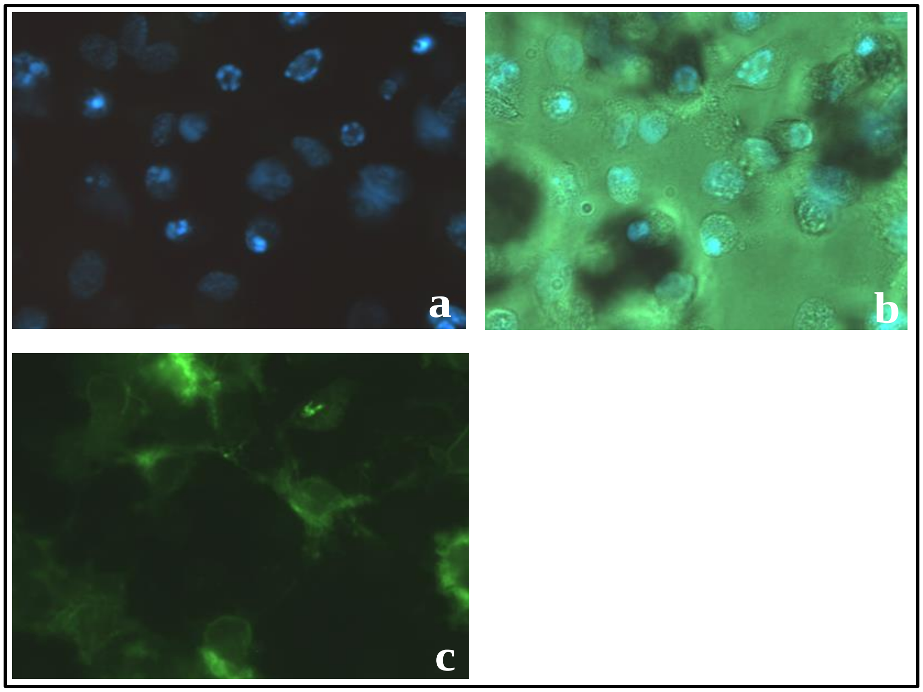

Figure 2. Effect of staurosporine on epithelial cells. Nuclei stained with DAPI (a), the bright field (b) and microfilaments decorated with FITC-phalloidin (c) is shown. Notice that fragmentation of nuclei in the cells is remarkable.
